# In utero exposure to endogenous maternal polyclonal anti-Caspr2 antibody leads to behavioral abnormalities resembling autism spectrum disorder in male mice

**DOI:** 10.1038/s41598-020-71201-9

**Published:** 2020-09-02

**Authors:** Ciara Bagnall-Moreau, Patricio T. Huerta, Davide Comoletti, Andrea La-Bella, Roseann Berlin, Chunfang Zhao, Bruce T. Volpe, Betty Diamond, Lior Brimberg

**Affiliations:** 1 Center for Autoimmune Musculoskeletal and Hematopoietic Diseases, Institute of Molecular Medicine, Feinstein Institutes for Medical Research, 350 Community Drive, Manhasset, NY 11030 USA; 2 Laboratory of Immune and Neural Networks, Institute of Molecular Medicine, Feinstein Institutes for Medical Research, Manhasset, USA; 3 Department of Molecular Medicine, Zucker School of Medicine At Hofstra/Northwell, 350 Community Dr, Manhasset, NY 11030 USA; 4 Departments of Neuroscience and Cell Biology Robert Wood Johnson Medical School, Rutgers, The State University of New Jersey, New Brunswick, NJ 08901 USA; 5 School of Biological Sciences, Victoria University of Wellington, Wellington, 6140 New Zealand; 6 Laboratory of Functional Neuroanatomy, Institute of Molecular Medicine, Feinstein Institutes for Medical Research, Manhasset, USA

**Keywords:** Neuroimmunology, Autism spectrum disorders, Autoimmunity

## Abstract

The concept that exposure in utero to maternal anti-brain antibodies contributes to the development of autism spectrum disorders (ASD) has been entertained for over a decade. We determined that antibodies targeting Caspr2 are present at high frequency in mothers with brain-reactive serology and a child with ASD, and further demonstrated that exposure in utero to a monoclonal anti-Caspr2 antibody, derived from a mother of an ASD child, led to an-ASD like phenotype in male offspring. Now we propose a new model to study the effects of in utero exposure to anti-Caspr2 antibody. Dams immunized with the extracellular portion of Caspr2 express anti-Caspr2 antibodies throughout gestation to better mimic the human condition. Male but not female mice born to dams harboring polyclonal anti-Caspr2 antibodies showed abnormal cortical development, decreased dendritic complexity of excitatory neurons and reduced numbers of inhibitory neurons in the hippocampus, as well as repetitive behaviors and impairments in novelty interest in the social preference test as adults. These data supporting the pathogenicity of anti-Caspr2 antibodies are consistent with the concept that anti-brain antibodies present in women during gestation can alter fetal brain development, and confirm that males are peculiarly susceptible.

## Introduction

Autism spectrum disorder (ASD) affects 1 in every 58 children in the United States, and is 4 times more prevalent in boys than girls^1^. ASD is diagnosed based on the presence of stereotypic behaviors and impairments in social skills and communication. Recent research has emphasized the importance of the in utero environment as a contributing risk factor for ASD. An elegant study demonstrated that siblings born within inter-birth interval of 18 months or less after the birth of a child with ASD have a higher risk for ASD than siblings born after an interval of 4 years or longer time period^2^. Moreover, maternal half siblings of a child with ASD are more at risk for ASD than paternal half siblings^2^.

Several studies looking into maternal risk factors for having a child with ASD have highlighted the importance of infection^3–5^, microbiome^6–8^, and brain-reactive antibodies^9,10^. For instance, women with autoimmune diseases such as rheumatoid arthritis (RA), celiac disease or systemic lupus erythematous (SLE) have an increased risk for a child with ASD^11,12^. This association holds if the mother (but not the father) exhibits the autoimmune disease. Since autoimmunity is characterized by autoantibodies, it has been hypothesized that maternal antibodies can pose a risk for ASD.

Maternal immunoglobulins of the IgG isotype begin to cross the placenta and enter the fetal circulation at the beginning of the second trimester of pregnancy (for review see, Ref.^13^). Since the blood–brain barrier (BBB) is not fully formed in the developing fetus, maternal IgG present in fetal circulation can penetrate fetal brain parenchyma^14^. Thus, if the mother has antibodies that target fetal brain antigens, these antibodies might affect brain development, while the mother will not be affected since she has mature and functional BBB.

A growing number of reports^10,15,16^, including our own^9^, have demonstrated that mothers of an ASD child are more likely to harbor anti-brain antibodies than mothers of a typically developed child or unselected women of child bearing age. Over 10% of mothers of an ASD child harbor antibodies against brain antigens, while such antibodies are found in only 2.5% of unselected women of child bearing age^9^ or in mothers of a typically developed child^17^.

Experiments in mice and non-human primates have shown adverse outcomes when brain-reactive serum or purified IgG derived from the mother of a child with ASD is transferred into pregnant females^15,16,18–20^. Braunschweig and colleagues identified intracellular antigens that are targeted by antibodies in the serum of mothers of an ASD child, and suggested that various combinations of these antibodies pose a risk for having a child with ASD^17^. More recently, Jones and colleagues showed that exposure in utero to polyclonal antibodies targeting a combination of peptides from LDH-A, LDH-B, STIP1 and CRMP1 (identified in Ref.^17^) induced some abnormal social and repetitive behaviors in female and male mice^21^.

We have isolated monoclonal IgG from mothers with anti-brain antibodies and an ASD child, and focused on a monoclonal antibody, termed C6, that binds to contactin-associated protein-like 2 (Caspr2)^22^, an extracellular protein expressed on neurons^23^, encoded by the CNTNAP2 gene. Importantly, we found that anti-Caspr2 IgG is present in ~ 40% of mothers with anti-brain antibodies and an ASD child^24^. While Coutinho et al.^25^ reported no excess of anti-Caspr2 antibodies in mothers of an ASD child, they did not first select for mothers with anti-brain antibodies. In a cohort of 95 mothers we would expect to have 9 mothers with anti-brain antibodies, of which 4 harbor anti-Caspr2 antibodies. In their study, they found one mother with anti-Caspr2 antibodies and with an ASD child. Their study was not designed to ask how often anti-Caspr2 antibodies are present in mothers with anti-brain antibodies and with an ASD child, but their data do not remarkably differ from ours.

Several roles for Caspr2 in neural development and synaptic connection have been uncovered recently, such as regulating neuronal migration^26^, glutamate receptor trafficking^27^, and facilitating the formation of new synaptic spines^28^. Caspr2 mutations in human pedigrees associate with neurologic abnormalities including ASD^29–31^, and deletion of the gene that encodes Caspr2 has been shown to lead to neurodevelopmental abnormalities in mice^26^.

Caspr2 expression in the brain begins during fetal development, and the level of expression is similar between females and males throughout gestation^24^. At gestational day E14, Caspr2 mRNA expression is localized to progenitor cells at the ventricular zone where excitatory projection neurons arise^23,26,32^. We have shown that gestating mice given C6 intravenously, on gestational day E13.5, have male offspring with an ASD-like phenotype, but normal female offspring^24^. Male mice exposed in utero to C6 displayed abnormal cortical development, decreased dendritic complexity of excitatory neurons and reduced numbers of inhibitory neurons in the hippocampus, as well as impairments in sociability, flexible learning, and repetitive behavior^24^. Caspr2 is considered to be expressed almost exclusively in the central nervous system^23^, and aside from its expression in the dorsal root ganglia^33^, it is sequestered behind the BBB. Anti-Caspr2 antibodies cannot penetrate the brain and therefore cannot cause brain insult with no compromise to the BBB. It should be mentioned that purified IgG containing anti-Caspr2 IgG from two male patients with neuropathic pain passively administered for 2–3 weeks to mice, resulted in hypersensitivity to provoked painful pressure suggesting that these patients’ antibodies produce pain sensitivity through the dorsal root ganglia sensory neurons^34^.

We were motivated to create a more physiologic model since we have found that about 40% of mothers with anti-brain antibodies harbor anti-Caspr2 antibodies^24^, and presumably they have a spectrum of antibodies reacting to different epitopes on Caspr2.

In the current study, we describe a model in which dams produce endogenous polyclonal anti-Caspr2 antibodies throughout gestation. Male, but not female mice, exposed in utero to polyclonal anti-Caspr2 IgG exhibit brain and behavioral alterations similar to our observation in mice exposed in utero to C6^24^ , and demonstrating that the male bias we previously observed does not reflect the time of exposure to anti-Caspr2 antibody.

The development of an immunization model in which antibodies are present throughout gestation permits an analysis of the progression of neurodevelopmental abnormalities and allows us to discern the window of time in which neurons are vulnerable to maternal anti-Caspr2 antibodies and susceptible to therapeutic strategies in future studies.

## Results

### Female mice immunized with Caspr2 developed high titers of IgG to mouse Caspr2

**Figure 1:**
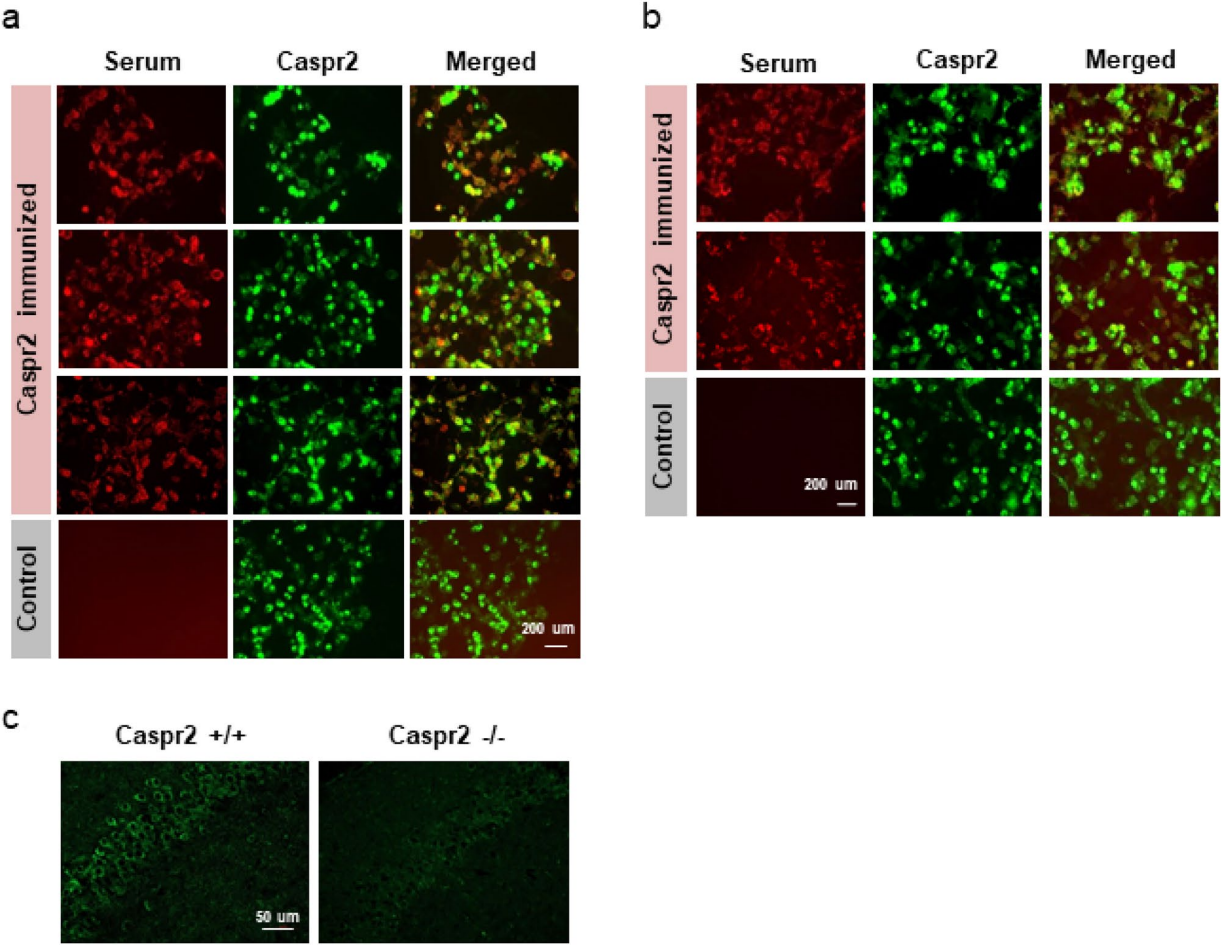
Serum from Caspr2 immunized mice binds Caspr2. (**A**) IgG from serum of Caspr2 immunized mice, but not from Control mice, co-localized with human (upper panel) and mouse (second panel) Caspr2 on HEK 293T, and on HEK GnTI- (third panel) cells expressing turbo-green florescent protein (tGFP)-Caspr2. Serum of Control immunized mouse showed no binding (bottom panel). No staining was seen on cells expressing only tGFP or non-transfected cells (data not shown). (**B**) Serum from E18.5 fetuses of Caspr2-immunized mice bound both human (upper panel) and mouse (middle panel) Caspr2 measured by a cell based assay. Serum from Control immunized mice showed no binding (lower panel). (**C**) Serum from Caspr2-immunized mice show reduced staining in the hippocampal CA1 region of Caspr2^−/−^ compared to Caspr2^+/+^mice.

Female mice immunized with the extracellular region of human Caspr2 developed high IgG titers to both human and mouse Caspr2 measured 2 weeks after the last boost (at the time of mating). Titers remained high until the time of weaning of the litter (6–8 weeks after the last boost) as determined by cell-based assays (Fig. 1A, Fig S1). Reactivity to both human and mouse Caspr2 was also observed in blood of E18.5 fetuses of dams immunized with Caspr2 but not in Control (CFA alone) immunized mice or in their fetuses (Fig. 1A,B, Fig S1). Given that the Caspr2 amino acid sequence is highly conserved between human and mouse, with 94% amino acid sequence identity in the extracellular region, the use of human Caspr2 as the immunogen ensured that self-tolerance to Caspr2 would be broken. Caspr2-specific B cells responding to the human protein function as antigen-presenting cells enabling reactivity to mouse Caspr2 to develop, as was described in a study of reactivity to self-cytochrome C^35^.

Serum of mice immunized with Caspr2 showed similar reactivity to Caspr2 expressed both on HEK293T and glycosylation deficient HEK293 GnTI– cells (Fig. 1A). Given that Caspr2 is highly glycosylated^36^, this observation ensured that antibodies were not specific for sugar moieties. In addition, serum from mice immunized with Caspr2 showed a reduced binding, if any, to brains of mice lacking Caspr2 compared to brains of wild-type mice (Fig. 1C). Both Control and Caspr2 immunized mice showed similar increase in serum IgG two weeks after the last boost compared to baseline (Fig S1) and a similar level of the proinflammatory cytokines, IL6, IL17a, IL23, and TNF-α (Mann Whitney, U = 4, P > 0.3). Control immunized mice showed a non-significant increase in IFN-Ɣ (Mann Whitney, U = 1, P = 0.06). The similar immune profile suggest that Complete Freund’s Adjuvant by itself leads to an increase in antibody level, and further indicates that further alterations reported are specific to anti-Caspr2 antibody exposure.

### *Cortical abnormalities following exposure *in utero* exposure to anti-Caspr2 IgG*

**Figure 2:**
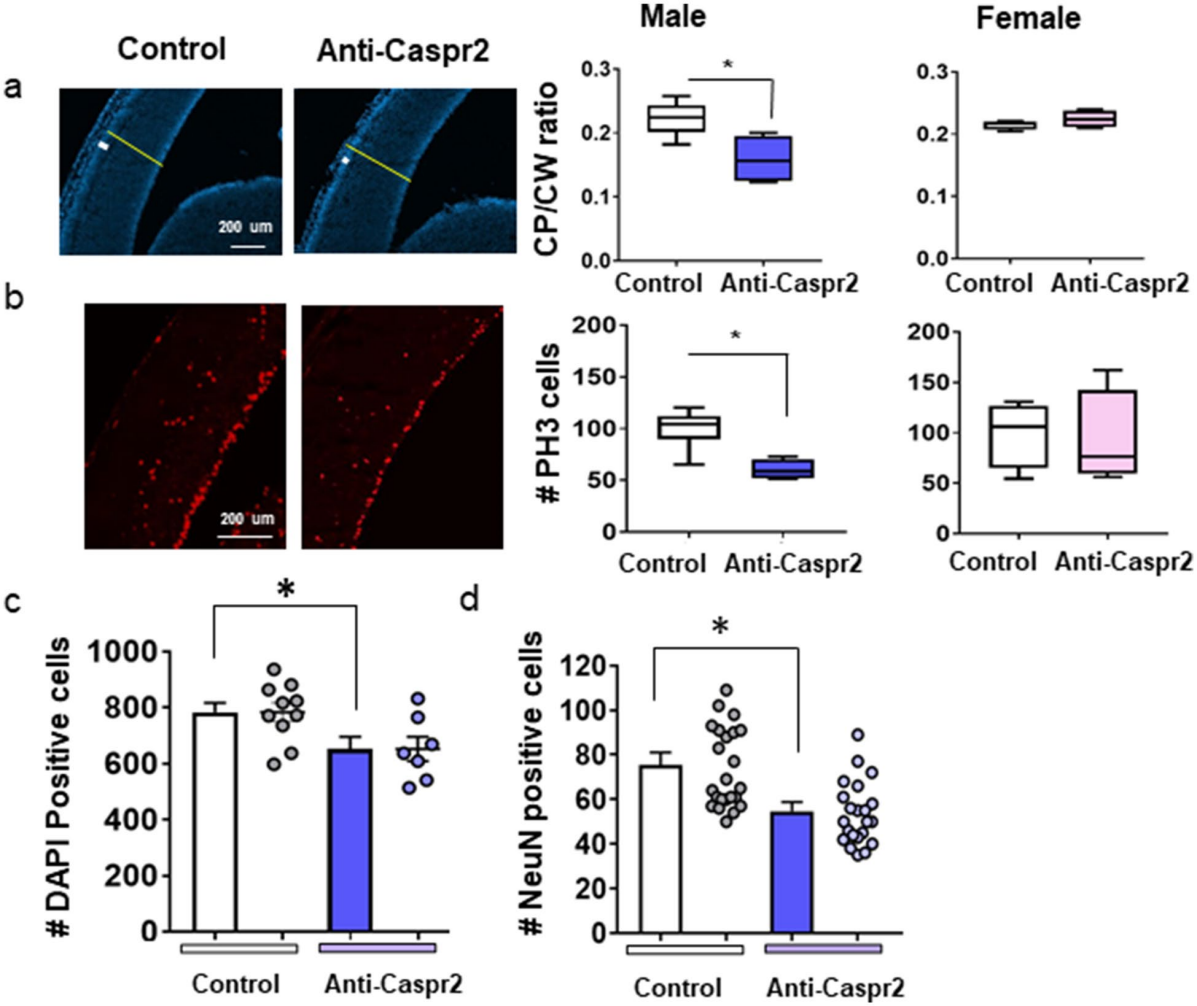
Male mice born to dams immunized with Caspr2 show cortical abnormalities. (A-B) Male fetuses (E15.5) from dams immunized with Caspr2 (Anti-Caspr2) display (**A**) a reduced ratio of cortical plate (CP) to cortical width (CW) and (**B**) a decreased number of proliferating cells in the ventricular zone compared to male fetuses of Control dams. (**A**) Left, DAPI staining. White and yellow lines indicate CP, and CW, respectively. Right**,** quantification of the ratio of CP to CW. Mann Whitney, Male U = 1 *P < 0.05, Female U = 5, n.s. (**B**) Left, PH3 + staining of mitotic cells. Right, quantification of PH3 + . Mann Whitney, Male U = 1 *P < 0.05, Female U = 7, n.s (**A**,**B**) Male, Control n = 6, Anti-Caspr2 n = 4; Female, Control, Anti-Caspr2, n = 4; 3 litters each. (**C**) Anti-Caspr2 male fetuses (E15.5) display a reduced number of DAPI positive cells across the cortex. Control n = 10, Anti-Caspr2 n = 7, 3 litters each. t-test, t(15) = 2.433, * *P* < 0.05. (**D**) Analysis of the average of counts from 2 to 4 sections per male mouse of NeuN positive neurons in the deep layers of the entorhinal cortex . Dots represent number of NeuN positive cells counts in a section. Control n = 7, 5 litters, Anti-Caspr2 n = 7, 4 litters. t-test, t(12) = 2.929, *P < 0.05.

Male, but not female, fetuses of dams harboring anti-Caspr2 antibody showed brain abnormalities including a reduced number of proliferating cells and thinning of the cortical plate (Fig. 2A,B) similar to male mice exposed in utero to C6 antibody^24^. Importantly, such abnormalities were not evident when the same experiment was performed in Caspr2 null mice (Fig. S2), although elevated titers of anti-Caspr2 antibody were observed in the dams after immunization and were similar to those induced in wildtype mice (Fig. S2). In line with reduced number of proliferating cells and the thinning of the cortical plate, we found an overall reduced number of DAPI + cells in the cortex of E15.5 male mice exposed in utero to anti-Caspr2 IgG (Fig. 2C) indicating a reduced number of neurons overall.

Adult male mice exposed in utero to anti-Caspr2 IgG showed cortical changes similar to what we reported for mice exposed in utero to C6^24^, specifically a reduced number of NeuN + neurons in the deeper layers of the entorhinal cortex (Fig. 2D). A gross examination of upper layers neurons did not reveal alterations in upper layers neurons.

### Alterations in excitatory and inhibitory neurons in the hippocampus

**Figure 3:**
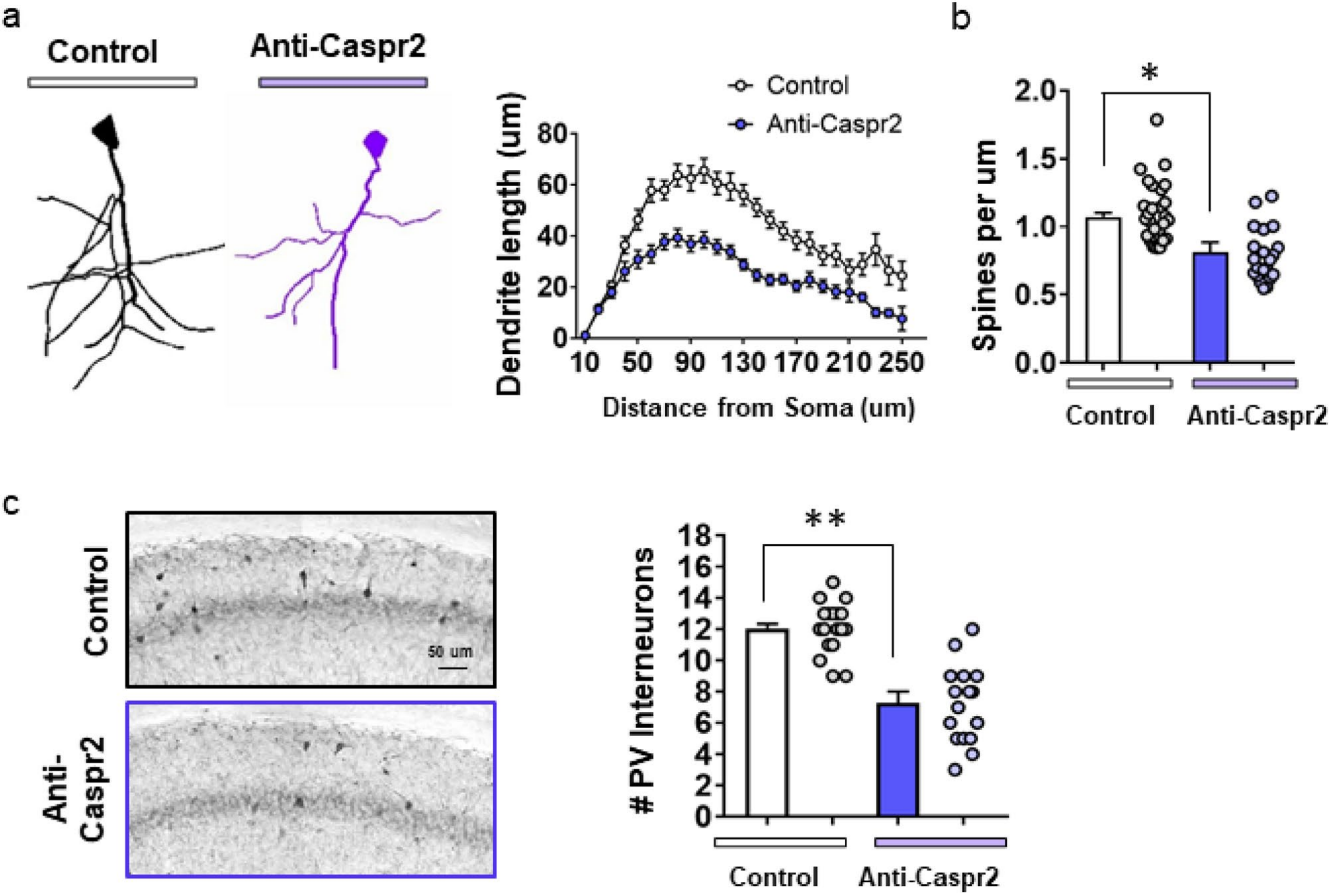
Male mice born to dams immunized with Caspr2 showed reduced dendritic arborization in pyramidal neurons and a reduced number of parvalbumin (PV) GABAergic interneurons. (**A**) Analysis of dendritic complexity in adult male mice exposed in utero to anti-Caspr2 (Anti-Caspr2, n = 5, 3 litters) or Control IgG (Control, n = 5, 4 litters). Left, tracings of CA1 neurons using Neurolucida360 (MBF, Williston VT), visualized with the Golgi method of silver staining. Right, Sholl analysis, male mice exposed in utero to Anti-Caspr2 IgG show decreased length of dendrites. Number of neurons = 44 per group. Mixed model linear analysis, *P* < 0.0001, ICC = 8%. (**B**) Analysis of the average number of synaptic dendritic spines per length in each mouse show reduced density of spines in CA1 neurons in mice exposed in utero to anti-Caspr2 IgG. Dots represent individual dendrites from which spines were counted, t-test, t(11) = 3.389, * *P* < 0.01. (Control, Anti-Caspr2, n = 4, 3 litters per group). (**C**) Analysis of the average counts of PV + GABAergic interneurons in the CA1 region. Dots represent number of individual PV + per section. t-test, t(9) = 6.639, * *P < 0.001, (Control n = 6, Anti-Caspr2 n = 5, 4 litters).

Since mice exposed in utero to C6 antibody showed reduced dendritic complexity^24^, we assessed pyramidal hippocampal neurons in mice exposed in utero to polyclonal anti-Caspr2 IgG, and observed fewer dendritic arborizations and spines in the neurons of male mice compared to mice exposed in utero to Control IgG (Fig. 3A,B).

We also stained for GABAergic PV + neurons in adult male mice exposed in utero to anti-Caspr2 or Control IgG, as mice exposed in utero to C6 antibody^24^ and mice with a genetic deletion of Caspr2^27^ display fewer GABAergic neurons. We found a reduced number of PV + neurons (Fig. 3C) in the hippocampus of male mice exposed in utero to anti-Caspr2 IgG.

We did not find differences in hippocampal pyramidal neurons and in PV + neurons between female mice exposed in utero to anti-Caspr2 or Control IgG (Fig. S3).

### *Behavioral abnormalities following exposure *in utero* exposure to anti-Caspr2 IgG*

Overall we found no difference between 5–7 weeks old male or female mice exposed in utero to anti-Caspr2 or Control IgG, when assessing body weight, coat, grip strength, body tone, reflexes, locomotion and sickness behavior as described in Ref.^24^ (Table S2). We did find that male mice exposed in utero to anti-Caspr2 IgG showed more rapid escape in response to light stoke in comparison with Control mice (“touch escape”, Table S2). However, in the open field test, avoidance of the center of the arena (suggestive of anxiety-like behavior) and total distance traveled (mobility) were not different between the two cohorts (t test, t (39) = 0.85, P > 0.4), at 8–10 weeks of age. We also did not find differences between the two cohorts in the rotarod test (Repeated measures ANOVA, Immunization × Trials, Interaction: F (4, 72) = 0.3, P > 0.8), suggestive of intact motor coordination and balance.

**Figure 4:**
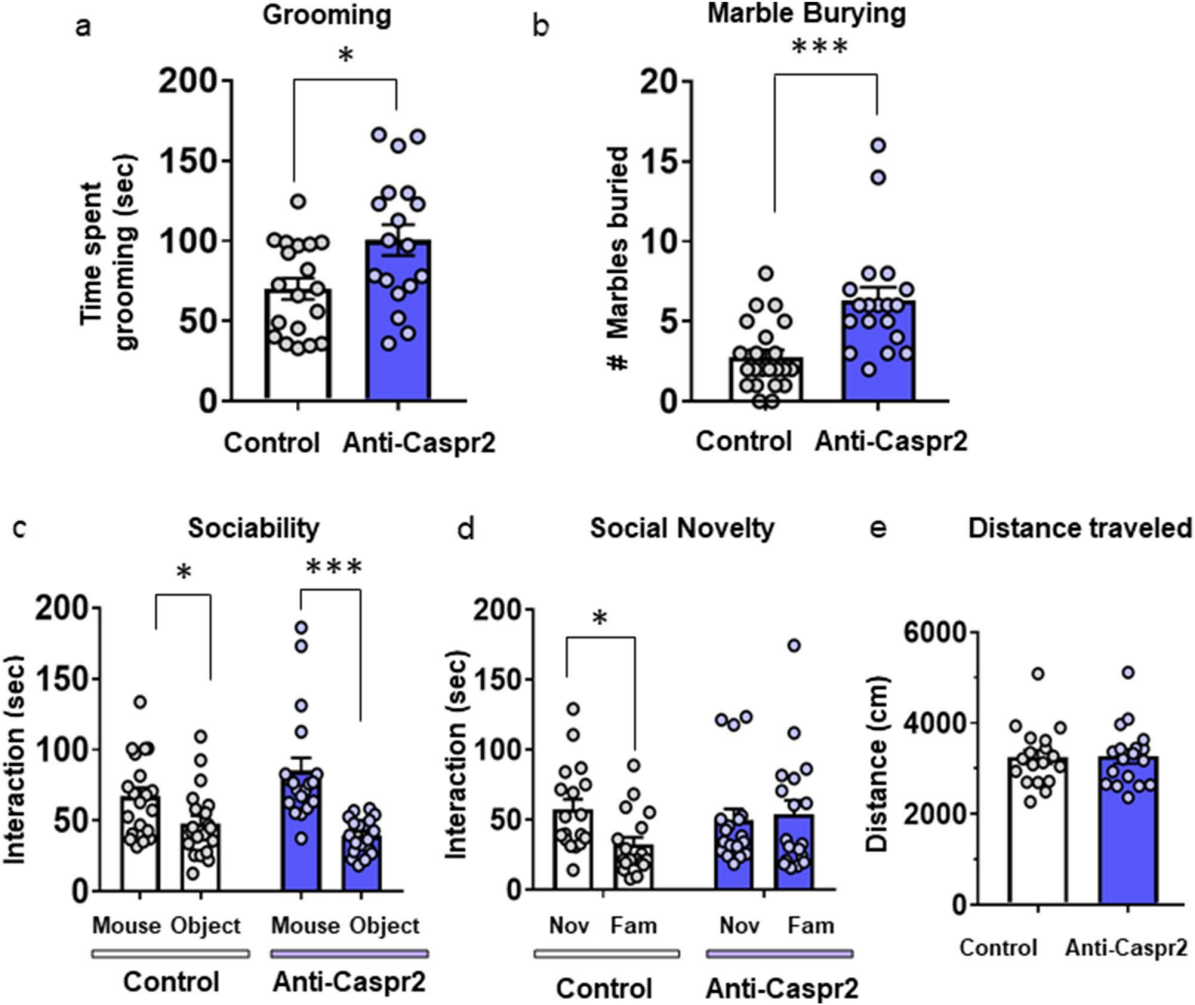
Behavioral phenotype of male mice born to dams harboring anti-Caspr2 IgG. (**A**) Grooming. Time spent grooming was recorded during two independent 15 min sessions and scored automatically using Ethovision software. Male mice born to Caspr2 immunized dams (Anti-Caspr2) spent overall more time grooming than male mice born to Control dams (Control). Control n = 19, Anti-Caspr2, n = 18, t-test, t(35) = 2.628, *P < 0.05. (**B**) Marble burying task. Anti-Caspr2 male mice buried more marbles than Control. Control, n = 22, Anti-Caspr2, n = 19, Mann Whitney, U = 66.5. ***P < 0.0001 (**C**) Sociability test. Control and Anti-Caspr2 offspring displayed a normal sociability defined as spending more time sniffing a mouse compared to an object. Control n = 20, Anti-Caspr2, n = 19. Two way repeated measures ANOVA, Immunization x Sociability Interaction: F (1, 37) = 7.828, *P* < 0.05, followed by Bonferroni post hoc test *P < 0.05, ***P < 0.0001. (**D**) Social Novelty test. Control offspring displayed a normal social novelty defined as spending more time sniffing a novel (Nov) mouse compared to a familiar (Fam) mouse, whereas offspring from Caspr2 immunized mice spent a similar time sniffing the two mice. Control n = 18, Anti-Caspr2, n = 18. Two way repeated measures ANOVA, Immunization × Novelty Interaction: F (1, 34) = 5.508, *P* < 0.05, followed by Bonferroni post hoc test *P < 0.05. (**E**) Distance traveled during the social Novelty test. Control and Anti-Caspr2 offspring did not show differences in total distance traveled during the social preference test Control n = 18, Anti-Caspr2, n = 18. t-test, t (34) = 0.07608, n.s. (**A**–**E**) Control, 9 litters, Anti-Caspr2, 7 litters, from two independent experiments. *P < 0.05, ***P < 0.001. Mean ± SEM.

We measured the time mice spent grooming and the number of marbles buried as assessments of a repetitive behaviors^37,38^. Male mice exposed in utero to anti-Caspr2 IgG spent more time grooming (Fig. 4A) and buried significantly more marbles than mice exposed in utero to Control IgG (Fig. 4B).

For the 3-chamber test, in the sociability phase, male mice of both groups showed increased interest in a novel mouse compared to the object (empty cylinder) (Fig. 4C). In the social novelty phase, mice exposed in utero to Control IgG spent more time sniffing a novel mouse versus a familiar mouse, whereas mice exposed in utero to anti-Caspr2 IgG did not show this social preference and spent a similar amount of time sniffing the novel and the familiar mouse (Fig. 4D). It should be noted that there was no difference in activity between groups, as the total distances moved during the social preference test were similar for the two groups (Fig. 4E).

Female offspring exposed in utero to anti-Caspr2 performed similarly to female mice exposed in utero to Control IgG in all assessments, further confirming the absence of a detectable insult to the developing female fetal brain (Fig. S4).

## Discussion

In the current study, male mice exposed in utero to polyclonal anti-Caspr2 antibody throughout gestation exhibited a pattern of behavioral abnormalities including repetitive behavior and social deficit that resemble an ASD–like phenotype. The current study shows that the male bias does not reflect administration of the C6 antibody at a time at which the male brain might be particularly vulnerable. Moreover, prenatal exposure to anti-Caspr2 antibody resulted in structural abnormalities in the cortex and the hippocampus. These results are similar to what was observed in mice following exposure in utero to the monoclonal anti-Caspr2 antibody, C6^24^. We previously demonstrated that C6, cloned from a mother of a child with ASD and administered on gestational day E13.5, elicited structural brain abnormalities and led to ASD-like behavioral impairments in male offspring^9^. Our current model is based on in utero exposure to endogenous polyclonal anti-Caspr2 following immunization. There was no variation in phenotype by litter suggesting that all polyclonal responses to Caspr2 harbor pathogenic responses. This is of special interest since we have found that about 40% of mothers with anti-brain antibodies harbor anti-Caspr2 antibodies^24^, and presumably they have a spectrum of antibodies reacting to different epitopes on Caspr2. We believe this model captures the human condition in which anti-Caspr2 antibody is present continuously during gestation. Using this model we will be able to investigate the effects of maternal anti-Caspr2 antibodies from the time they are crossing the placenta to the time the BBB prevents the penetration of maternal antibodies to the developing brain.

Most studies in mice and rhesus monkeys investigating a link between ASD and in utero exposure to maternal antibody have employed a passive antibody approach, exploring the effect of IgG pooled from serum of women with brain-reactive antibodies and a child with ASD^18,20,39^. One of these studies showed that passive transfer of IgG containing anti-Caspr2 antibody to murine dams led to social deficits, loss of glutamatergic synapses and microglial activation in male and female offspring^40^. This study confirmed the pathogenicity of in utero exposure to anti-Caspr2 antibody, although the IgG was purified from two elderly male patients with encephalitis and anti-Caspr2 antibody.

There is only one study of offspring exposed in utero to endogenous production of ASD-relevant antibody. Jones and colleagues^21^, immunized female mice with 21 peptides, each 15–20 amino acids long, corresponding to the sequence of intracellular proteins (LDH-A, LDH-B, STIP1 and CRMP1) previously suggested to be targets of antibodies in the serum of mothers of an ASD child^17^. Male and female mice offspring showed increased grooming as well as social interaction deficits during dyadic play but not in the 3 chamber sociability test.

In utero exposure to anti-Caspr2 IgG resulted in structural abnormalities in the brain of male mice, which persisted into adulthood. We noted a reduced number of neurons in the deeper layers of the entorhinal cortex. Alterations to deep layer pyramidal neurons have been reported in disparate ASD models^19,41,42^. Layers 5 and 6 start to develop by E12.5^43^, a time when the embryonic brain is accessible to IgG in the circulation^13^. The deep layer pyramidal neurons express Caspr2^23^, and in the entorhinal cortex they are positioned uniquely to integrate inputs from the hippocampus and communicate with other cortical areas^44^. Therefore, numerous brain circuits and associated behaviors may also be disrupted by anti-Caspr2 IgG. Moreover, we found that exposure to anti-Caspr2 IgG resulted in both loss of GABAergic PV + inhibitory neurons and reduced dendritic arborization and spines in CA1 excitatory neurons. Various ASD mouse models including Caspr2 null mice demonstrate abnormal hippocampal synaptic function^26,33,45,46^ including changes in both inhibitory and excitatory neurons^26–28,42^.

In utero exposure to anti-Caspr2 IgG produced several behavioral abnormalities in adult male mice. Interestingly, while exposure in utero to monoclonal anti-Caspr2 IgG resulted in abnormal sociability (i.e., preference toward object rather than a novel mouse) in a Y maze, in the current model, mice exposed in utero to anti-Caspr2 IgG showed alterations in social novelty (i.e., preference for a novel mouse over a familiar mouse) in the 3-chamber test. It is not clear if the difference is a response to the different anti-Caspr2 specificities (polyclonal versus monoclonal antibody) or a change in the time of exposure with earlier exposure in the polyclonal model. The lack of discrimination between social targets in the social novelty test may indicate impairment in social memory^47^. It has been shown that hippocampal neurons mediate social memory^48^, and that neurons projecting from the cortex to the hippocampus can affect social memory without affecting sociability^49^. Understanding the differences in these models requires further study.

Mice exposed in utero to polyclonal anti-Caspr2 IgG showed repetitive behaviors similar to mice exposed in utero to monoclonal anti-Caspr2 IgG^24^. While the hippocampus has been associated with social behavior^47,48^, the relationship between the hippocampus and repetitive behaviors is less clear. The hippocampus has connections to several brain regions in cortico-basal ganglia-thalamic circuits, including the striatum^50^, suggesting the possibility that neurons in the hippocampus may affect this circuit to modulate repetitive behaviors.

As in our previous model^24^, we found that male mice were specifically affected by exposure in utero to maternal anti-Caspr2 IgG. Gender specific neurodevelopmental effects have been documented in a mouse model of lupus, in which maternal anti-DNA antibodies cross reactive with the NMDA receptor leads to death of female fetuses and cognitively impaired male offspring. We did not find differences in the level of circulating anti-Caspr2 IgG in serum of male and female fetuses, and Caspr2 is expressed at similar levels in male and female fetuses on E15.5, and at postpartum^24^. Hormonal differences between male and female fetuses begin as early as E12.5, and may exert a protective effect in female mice exposed in utero to anti-Caspr2 IgG, acting either at the time of the exposure to antibody or initiating a compensatory program at a later time point. Consistent with this hypothesis, it has been shown that estrogen can reverse the behavioral phenotype in CNTNAP2 zebrafish mutants^51^.

It should be noted that while this study confirms the pathogenicity of anti-Caspr2 antibodies, and further suggest that the monoclonal C6 antibody does not function through an unrecognized cross-reactivity, we have not yet determined the precise mechanism by which maternal anti-Caspr2 antibodies are causing brain and behavioral alterations. One possibility is that anti-Caspr2 antibodies may create a functional hypomorph of Caspr2^24^ at a critical moment in brain development. This is supported partially by some similarity between the current model and the phenotype of Caspr2 null mice^26,33^. However, in our model, anti-Caspr2 antibodies might act on Caspr2 transiently as opposed to the permeant deletion of Caspr2 in Caspr2 null mice. The second possibility is that anti-Caspr2 immune complexes activate microglia through binding activating Fc receptors. A previous study has shown that exposure in utero to IgG purified from plasma of patient with high titers to Caspr2 resulted in increased activation of microglia^40^. Anti-Caspr2 antibody on the membrane of neurons may recruit C1q to permit dendritic pruning. Interestingly, C1q-tagged neurons have decreased levels of Neuronal pentraxin 1 (Nptx1), a protein involved in AMPA receptors trafficking^52^. Impaired AMPA receptor trafficking can affect dendrites and spines morphology^53^. Alternatively, C1q decorated synapses may be targeted for pruning^54^. These hypotheses require further studies.

## Methods

### Animals

Animal use was in accordance with institutional guidelines of the Feinstein Institutes for Medical Research. All protocols were approved by the Feinstein Institutes Animal Care and Use Committee (IACUC). C57BL/6 and B6.129(Cg)-Cntnap2tm1Pele/J (Caspr2 null) mice (5 weeks old) were obtained from the Jackson Laboratory and were allowed to acclimate to the animal room for one week. Six week old female mice were randomly assigned to be immunized with Caspr2 or adjuvant alone (Control). Female mice were immunized intra-peritoneal (i.p.) with 50 ug (in 50 μl of saline) of the extracellular region of human Caspr2 (Caspr2 1,261,^36^) expressed using glycosylation deficient HEK293T GnTI − cells^36^ in Complete Freund’s Adjuvant (50 μl) on day 1, followed by i.p. booster injections of 50 ug (in 50 μl of saline) of immunogen mixed with 50 μl of Incomplete Freund’s Adjuvant on days 14 and 28. Control female mice were immunized with 50 μl of saline mixed with 50 μl of adjuvant. Two weeks after last boost, immunized mice were mated with naïve males.

For fetal brain analysis timed pregnancies were generated by housing 2 females from the same treatment and same genotype and 1 non-treated male (with a matching genotype) together for 14 h. The time when the male mouse was removed from the cage was designated embryonic (E) day 0.5. Embryos were harvested at E15.5, or E18.5 and processed for sex identification and fetal brain pathology (E15.5)^24^, or blood collection (E18.5). Additional pregnancies were allowed to reach full term for behavioral and histology assessments of offspring during adulthood.

Table S1 specifies the number of mice per litter that were used in each test.

### Blood collection

Blood was collected by cheek bleed from immunized mice at baseline (right before immunization), 2 weeks after last immunization (before mating), and at the time embryos were harvested or when offspring were weaned. Blood samples were allowed to sit at room temperature for 30 min. They were then centrifuged for 5 min at 10,000 RPM, twice. Serum was collected and stored at − 20 °C.

### IgG ELISA

We determined the amount of mouse IgG in the serum of immunized mice at time 0, and 2 weeks after last immunization by ELISA as described in^24,55^. In short, plates coated with goat anti-mouse IgG (ThermoFisher Scientific, 62-6500) were incubated with serial diluted serum starting at 1:2,500 for 1 h at room temperature (RT). After washing, AP labeled goat anti-mouse IgG diluted 1:1,000, (ThermoFisher Scientific, 31320) was added to the plates at RT for 1 h. The assay was developed at RT using phosphate substrate tablets (Sigma, S0942).

### Cytokine assays

Cytokine analysis was performed on 8 maternal Caspr2 and Control immunized serum pulled to 4 samples each, using a custom U-PLEX Biomarker kit (Meso Scale Discovery (MSD), Rockville, MD), including U-PLEX Mouse IFN-γ, U-PLEX Mouse IL-6, U-PLEX Mouse IL-17A, U-PLEX Mouse IL-23 and U-PLEX Mouse TNF-α. MSD plates were analyzed on the MS2400 imager (MSD). The assay was performed according to the manufacturer’s instructions. All standards and samples were measured in duplicate.

### Binding assays using transfected HEK-293T cells

Serum of immunized mice and fetuses was analyzed for anti-Caspr2 IgG using a live cell-based immunofluorescence assay as previously described^24^. Antibody titers against Caspr2 were assessed against both human and mouse Caspr2 as described in^24^ using HEK-293 T or HEK293T GnTI-cells (ATCC).

### Immunohistochemistry of fetal brains

Sections were prepared as described in^24^. E15.5 brain sections were stained with Phospho-Histone 3 (PH3) and DAPI, as described in^24^. In short, tissue sections were blocked for 1 h with PBS (5%) with bovine serum albumin (BSA) in Triton X100 (0.1%) at RT. Anti PH3 antibody (1:100, Millipore 06-570) and DAPI (1 µg per ml, Life Technologies) were added overnight at 4 °C. After washing in PBS/0.1%Tween, antibody binding was detected using Alexa 594 goat anti-rabbit (Life Technologies) and visualized with an Axio-Imager (Z-1, Axio-Vision 4.7, Zeiss).

The number of proliferating cells (PH3 + cells) in the ventricular zone was quantified by automat image analysis programs on the Axio-Imager microscope. We identified anatomical regions using within-section coordinates and counted positive cells in comparable ventricle zone area^24^.

The ratio for cortical plate (CP) over cortical width (CW), termed CP/CW, was evaluated automatically by lab-made software (Gata-Gracia et al., Under Review). In short, CP/CW ratio was calculated by repeated sampling throughout the length of the defined cortical plate. Mean values of CP/CW ratios were used for comparison.

E15.5 brains were also evaluated for total DAPI + cells, which were counted using the Fiji image processing package of Image J. Images (100 × 100 μm) were subdivided into 10 equal bins to segment the lamination of the cortex avoiding the bias of identifying manually brain layers. A threshold and watershed algorithm was applied to segment and define the cell clusters in the image. Cells were automatically counted within each bin as defined by size and sphericity using the ‘analyze particle’ function. A summary of the total number of positive cells counted was analyzed.

### Immunohistochemistry of adult brains

Sixteen to 20 week old mouse brains were prepared for NeuN labeling (anti-NeuN antibody, Millipore, Mab 337), parvalbumin (PV) labeling (Anti-PV antibody, Abcam, ab11427), and Golgi method as described in Ref.^24^.

The number of NeuN + neurons was determined in the deep layers (layers 5–6) of the entorhinal cortex, modifying the nearest neighbor approach that was formerly used (MBF, Williston, Vt.^24^). A systematic random sampling grid was positioned ventral and medial to the rhinal notch (Bregma − 3.52 mm), with upper structured layers as anchor points. Neurons in focus in the optical planes within the sampling grid were counted. Each run represents the neuron number of an individual section. There were 2–4 sections per mouse.

We counted PV + neurons from matched coronal sections (40 µm thick) across the stratum pyramidale of CA1 region of the dorsal hippocampus (Bregman − 1.20 to − 1.80 μm). Identical volumes were sampled (0.191 mm^2^) at 20 × (Axio-Imager Z1; Zeiss), and 2–4 sections were counted per mouse.

Quantification of dendrites and spines was performed on Golgi stained sections (FD Rapid GolgiStain Kit, Ellicott City, MD) as described in detail in Refs.^24,56^. Briefly, for dendrite analysis, images were collected (40 ×; Axio-ImagerZ-1, tiled, z-stack 2.0 µm) of neurons in the stratum pyramidale of the CA1 hippocampus (comparable sections, 100 µm), approximately one of four periodicity from Bregma − 0.94 to 2.0 mm that had apical dendrites and a cell body and were distinguishable from nearby neurons. Dendrites were traced, and a Sholl template was imposed on the trace (concentric shells of increasing diameter) in order to measure the total dendritic length for each shell (Neurolucida360, MBF, Williston VT)^57^. While the mean dendritic length for each shell is displayed, all the measurements were analyzed using a mixed model linear analysis in order to account for the clustering of dendrite measures. We evaluated whether there was a difference in outcome between mice exposed in utero to anti-Caspr2 or Control IgG, while accounting for three-level hierarchical design, where the unit of observation was the radius of a shell that was nested within cells, which were nested within mice.

For the spine analysis, images were collected of dendrites of CA1 pyramidal neurons (at 100 ×: Axio-Imager Z1, tiled, Z-stack 0.5-μm). Spines were visualized and counted (per µm of dendritic length).

### Behavioral assessments

A primary behavioral screen was performed at 5–7 weeks of age on mice that were exposed in utero to anti-Caspr2 or Control IgG, and this was followed by behavioral tests at 8–14 weeks of age. Mice were maintained on a reverse schedule of dark (09:00 to 21:00) and light (21:00 to 9:00) with ad libitum access to food and water. Mice undergoing behavioral assessments were analyzed according to their cage number, which did not indicate the in utero exposure to antibody; therefore, the testing was performed in a blinded fashion. Female and male mice were tested on separate days. One week before testing, each mouse was handled for 3 days in 15 min sessions during the dark period of their circadian cycle. A behavioral screen was conducted to assess autonomic responses and neurological reflexes^24^. Animal behavior was recorded with a centrally placed camera using video tracking software (EthoVision v14, Noldus, Attleboro, MA, USA).

The open field test, grooming behavior assessment and marble burying test were performed in a square arena (40 cm on each side) with dark walls (30 cm high). The open field test was used to examine mobility and the lack of occupancy of the center of the arena was defined as a measure of anxiety as described in Ref.^24^. Mice were examined for 15 min during the habituation phase. The next day, grooming behavior was recorded in two sessions (15 min each, 2 h apart) and analyzed automatically with the “mouse behavior recognition” module within EthoVision. The marble burying assay was performed as described in Ref.^24^ with slight modifications. Each individual mouse was placed in the arena with 4 cm deep bedding in which 25 black glass marbles (1.2 cm diameter) were placed in a 5 × 5 arrangement. The number of marbles buried in 20 min (> 50% of the marble covered by bedding material) was recorded. Marbles were washed with alcohol and water, dried and rubbed in fresh bedding to eliminate odors, between each animal.

The rotarod test was performed as previously described^58^. The rotarod was used to asses motor coordination and balance Briefly, each mouse was placed on a rotating drum (ENV-576 M, Med Associates Inc, St. George, VT, USA), with an acceleration from 4 to 40 rpm over 5 min. The time at which the mouse fell off the drum was recorded. The test was repeated 5 times for each mouse with an interval of at least 1 h between trials.

The 3-chamber social task was performed as described in Ref.^59^. This task exploits the natural tendency of mice to approach and investigate an unfamiliar mouse over an object or a familiar mouse^60^. In short, mice were placed within a 3-chamber arena (clear Plexiglas) with openings between the center and the side chambers in order to allow for free passage between them. Mouse behavior was tracked and recorded (Ethovision) during 3 consecutive phases (10 min each), with no longer than 2 min delay interval to allow the preparation for next phase. On phase 1 (habituation), the test mouse was placed in the middle chamber and allowed to explore the empty arena for 10 min. On phase 2 (sociability), an unfamiliar animal (mouse 1) of the same age, sex and genotype was placed in one of the two peripheral chambers within a cylinder that allowed for social interaction but prevented any escape or mounting behavior; a second (empty) cylinder was added in the opposite peripheral chamber. The cylinders were identical (diameter, 9 cm; height, 20 cm), with the bottom 5.5 cm perforated with holes (diameter, 0.5 cm), and were 3D printed using blue methacrylate resin (Form2 printer, FormLabs, Somerville, MA, USA). The sociability of the test mouse was assessed over the 10 min period by its willingness to explore mouse 1, as opposed to the empty cylinder. On phase 3 (social novelty), another unfamiliar mouse (mouse 2) of the same sex and genotype was introduced into the empty cylinder and was denoted as the ‘novel’ mouse, while mouse 1 from the previous phase was the ‘familiar’ mouse. The social novelty behavior of the test mouse was then measured by its preferential exploration of the novel mouse over the familiar mouse.

### Statistical analysis

We used Mann–Whitney test to compare medians of two independent groups for small datasets or for non-normally distributed samples, otherwise we used two-sample Student's t-tests.

We used a mixed model linear analysis in order to account for clustering of measures, and calculated intraclass correlation coefficient (ICC), to indicate a reasonable variance accounted for by clustering.

To analyze the rotarod test we performed a Repeated Measures ANOVA with a within subjected factor of trials and immunization (Anti-Caspr2/ Control) as the between subject factor.

We used similar analysis to analyze the three chambered social test with a within subjected factor of sociability or novelty and immunization (Anti-Caspr2/ Control) as the between subject factor, followed by Bonferroni post hoc test (the specific comparisons are demonstrated in the figures).

Tests involved two groups were analyzed using Graph Pad Prism 7. ANOVA was performed with the statistical toolbox of Origin v.11. Mixed model linear analysis was performed on R program 3.6.2. Values were considered significant for p < 0.05. Data are presented as mean and error bars represent the standard error or as median and range. All tests were performed two-tailed.

## Supplementary information

Supplementary file 1

## Data Availability

The datasets generated during the current study are available from the corresponding author upon request.
