# Supplementary material for: In utero exposure to endogenous maternal polyclonal anti-Caspr2 antibody leads to behavioral abnormalities resembling autism spectrum disorder in male mice
Source: Sci Rep. 2020 Sep 2;10:14446. doi: 10.1038/s41598-020-71201-9 (PMC7468145; doi:10.1038/s41598-020-71201-9)

Supplementary Material

In utero exposure to endogenous maternal polyclonal anti-Caspr2 antibody leads to behavioral abnormalities resembling autism spectrum disorder in male mice.

Authors: Ciara Bagnall-Moreau[a]; Patricio T. Huerta[b,c]; Davide Comoletti[d,e,f]; Andrea La-Bella[a]; Roseann Berlin [g]; Chunfang Zhao[a]; Bruce T [g]. Volpe, Betty Diamond[a,+] and Lior Brimberg[a+]*.

Affiliations:

[a] Center for Autoimmune Musculoskeletal and Hematopoietic Diseases, Institute of Molecular Medicine, Feinstein Institutes for Medical Research.

[b] Laboratory of Immune & Neural Networks, Institute of Molecular Medicine, Feinstein Institutes for Medical Research

[c] Department of Molecular Medicine, Zucker School of Medicine at Hofstra/Northwell, 350 Community Dr, Manhasset NY 11030, USA

[d] Departments of Neuroscience and Cell Biology Robert Wood Johnson Medical School, Rutgers, The State University of New Jersey, New Brunswick, NJ 08901, USA;

[e] Department of Pediatrics, Robert Wood Johnson Medical School, Rutgers, The State University of New Jersey, New Brunswick, NJ 08901, USA;

[f] School of Biological Sciences, Victoria University of Wellington, Wellington 6140, New Zealand.

[g] Laboratory of Functional Neuroanatomy, Institute of Molecular Medicine, Feinstein Institutes for Medical Research.

+Co Senior authors

*Corresponding Author

***Corresponding Author:**

Lior Brimberg

Center for Autoimmune Musculoskeletal and Hematopoietic Diseases,

Institute of Molecular Medicine, Feinstein Institutes for Medical Research,

350 Community Drive, Manhasset NY 11030, USA

lbrimberg@northwell.edu

Supplementary Figure legend:

Supplementary Figure 1: Dams immunized with Caspr2 and their fetuses harbor anti-Caspr2 IgG (A) No difference in fold IgG change from baseline (the day of first immunization) between Control (n=10) and Caspr2 (n=13) immunized mice. Mann Whitney, U=56, $P > 0.5$. (B) Serum from Caspr2 immunized mice show high titer reactivity to both human and mouse Caspr2 measured by a cell based assay. Serum from Control mice showed no binding. Upper panel, two weeks after last immunization, Lower panel, six weeks after last immunization. Control, Anti-Caspr2, n=13, Mann Whitney, U=0, ***$P < 0.0001$. (C) Serum from E18.5 fetuses of Caspr2 mice show reactivity to both human (left panel) and mouse (right panel) Caspr2 measured by a cell based assay. Serum from Control mice showed no binding. No differences were recorded between male and female offspring of dams immunized with Caspr2. (Control Male, n=11, Female n=13, 3 Litters, Anti-Caspr2, Male, n=12, Female n=13, 4 litters). Mann Whitney, U=0, ***$P < 0.0001$. (B+C) Titers denote dilution of serum leading to binding in a cell based assay.

Supplementary Figure 2: No cortical development abnormalities were observed between male fetuses exposed in utero to anti-Caspr2 or Control IgG in Caspr2 -/- mice. (A) Caspr2 -/- mice immunized with Caspr2 developed high titers to Caspr2 measured by cell based assay. Control, Caspr2 n=4. Mann Whitney, U=0, *$P < 0.05$. (B) Quantification of the ratio of CP to CW in offspring born to dams harboring Anti-Caspr2 or Control IgG. Control n=8, Anti-Caspr2 n=5, 2 litters. Mann Whitney, U=13, n.s (C) quantification of mitotic cells (PH3+). Control n=4, Anti-Caspr2 n=4, 2 litters Mann Whitney, U=4, n.s.

Supplementary Figure 3: Female mice born to dams immunized with Caspr2 showed no abnormality of dendritic arborization in pyramidal neurons and a similar number of parvalbumin (PV) GABAergic interneurons compared to Control females mice. (A) Analysis of dendritic complexity in adult female mice exposed in utero to anti-Caspr2 (Anti-Caspr2, n=5, 4 litters) or Control IgG (Control, n = 6, 3 litters). Left, tracings of CA1 neurons using Neurolucida360 (MBF, Williston VT) visualized with the Golgi method of silver staining. Right, Sholl analysis, female mice exposed in utero to Anti-Caspr2 IgG show similar length of dendrites compared to Control female mice. Number of neurons: Anti-Caspr2=37, Control=36. Mixed model linear analysis, P =0.287, ICC=9%. (B) Analysis of the average number of synaptic dendritic spines per length in each mouse show similar density of spines in CA1 neurons in female mice exposed in utero to anti-Caspr2 (Anti-Caspr2, n=5, 4 litters) or Control (Control, n = 6, 3 litters) IgG. Dots represent individual dendrites from which spines were counted, t-test,

t(9)=1.5, n.s. (C) Analysis of the average counts of PV+ GABAergic interneurons in the CA1 region. Dots represent number of individual PV+ per section. t-test, t(9)=0.6, n.s. (Anti-Caspr2, n=5, 4 litters, Control n=6, 3 litters).

Supplementary Figure 4: Behavioral phenotype of female mice exposed in utero to anti-Caspr2 or Control IgG. (A) Female mice born to Caspr2 immunized dams (Anti-Caspr2) were not different from female mice born to Control dams (Control) in time spending grooming. Time spent grooming was recorded during two independent 15 min sessions and scored automatically using Ethovision software .Control n=11, 4 litters, Anti-Caspr2, n=13, 5 litters, from one experiment, t-test, t(22)=0.9583, n.s. (B) Marble burying task. Control, n=23, 7 litters Anti-Caspr2, n=18, 7 litters, from two independent experiments. Mann Whitney, U=181.5, n.s. (C) Sociability test. Control and Anti-Caspr2 offspring displayed a normal sociability defined as spending more time sniffing a mouse compared to an object. (D) Social Novelty test. Control and Anti-Caspr2 offspring displayed a normal social preference defined as spending more time sniffing a novel (Nov) mouse compared to a familiar (Fam) mouse. (C+D) Two way repeated measures ANOVA with Bonferroni post hoc test, * $P < 0.05$, ** $P < 0.005$ Means $\pm$SEM. (E) Distance traveled during the Social Novelty test. Control and Anti-Caspr2 offspring did no show differences in total distance traveled during the social preference test, t-test , t(44)=0.2108, n.s. (C-E) Control n=21, 8 litters, Anti-Caspr2, n=24, 9 litters from two independent experiments.

Supplementary Table 1: Number of mice per litter

Figure #	Test	Immunization	# Litter	# of pups per litter	Total N
Fig. 2 A-B	CP/CW, PH3	Control	3	1-2	M =6 F=4
		Anti-Caspr2	3	1-2	M=4, F=4
Fig. 2 C	# DAPI$^+$ cells	Control	3	5,4,1	10
		Anti-Caspr2	3	4,2,1	7
Fig. 2 D	# NeuN$^+$ cells	Control	5	1-2	7
		Anti-Caspr2	4	1-2	7
Fig. 3 A	Golgi-Dendrites	Control	4	1-2	5
		Anti-Caspr2	3	1-2	5
Fig. 3 B	Golgi-Spines	Control	3	1-2	4
		Anti-Caspr2	3	1-2	4
Fig. 3 C	# PV$^+$ cells	Control	4	1-2	6
		Anti-Caspr2	4	1-2	7
N/A	Rotarod	Control	5	3,3,1,1,1	9
		Anti-Caspr2	3	4,4,3	11
N/A	Open Field	Control	9	4,4,3,3,3,2,1,1,1	22
		Anti-Caspr2	7	4,4,4,3,2,1,1	19
Fig. 4 A	Grooming	Control	9	4,3,3,3,2,1,1,1,1	19
		Anti-Caspr2	7	4,4,3,3,2,1,1	18
Fig. 4 B	Marble Burying	Control	9	4,4,3,3,3,2,1,1,1	22
		Anti-Caspr2	7	4,4,4,3,2,1,1	19
Fig. 4 C	Sociability	Control	9	4,4,3,3,2,1,1,1,1	20
		Anti-Caspr2	7	4,4,4,3,2,1,1	19
Fig 4 D-E	Social Novelty	Control	9	3,3,3,3,2,1,1,1,1	18
		Anti-Caspr2	7	4,4,3,3,2,1	18
Fig S1 C	Anti-Caspr2 IgG	Control	3	8,10,6	M =11 F=13
		Anti-Caspr2	4	4,5,8 8	M =12 F=13
Fig S2 A	CP/CW	Control	2	4,4	8
		Anti-Caspr2	2	2,3	5
Fig S2 B	PH3	Control	2	2	4
		Anti-Caspr2	2	2	4
Fig S3 A	Golgi-Dendrites	Control	3	2	6
		Anti-Caspr2	4	1-2	5
Fig S3 B	Golgi-Spines	Control	3	2	6
		Anti-Caspr2	4	1-2	5
Fig S3 C	# PV$^+$ cells	Control	3	4,1,1	6
		Anti-Caspr2	4	1-2	5
Fig S4 A	Grooming	Control	4	5,3,2,1	11
		Anti-Caspr2	5	4,4,3,1,1	13
Fig S4 B	Marble Burying	Control	7	5,5,4,3,3,2,1	23
		Anti-Caspr2	7	4,4,3,3,2,1,1	18
Fig S4 C	Sociability	Control	8	5,4,4,3,2,2,1	21
		Anti-Caspr2	9	4,4,3,3,3,3,2,1,1	24
Fig S4 D-E	Social Novelty	Control	8	5,4,4,3,2,2,1	21
		Anti-Caspr2	9	4,4,3,3,3,3,2,1,1	24

Supplementary Table 2: Primary screen

A. Male

No.	Variable	Scale Range	Control	Anti-Caspr2	*P*
1	Coat length	0–4	0 (0/0)	0 (0/0)	1
2	Hair length	0–3	0 (0/0)	0 (0/0)	1
3	Hair morphology	0–2	0 (0/0)	0 (0/0)	1
4	Body position	0–8	5 (4/5)	4 (4/5)	0.32
5	Spontaneous activity	0–8	4 (4/5)	4 (3/6)	0.068
6	Respiration rate	0–3	2 (2/2)	2 (2/2)	1
7	Tremor	0–2	0 (0/0)	0 (0/0)	1
8	Defecation	0–10	2 (0/4)	1 (0/2)	0.6
9	Urination	0–1	0 (0/1)	0 (0/0)	0.86
10	Transfer arousal	0–6	5 (0/5)	4 (2/5)	0.6
11	Latency to move	0–30	2 (0/3)	2 (0/4)	0.9
12	Locomotion	0–30	13 (8/20)	13 (9/26)	0.7
13	Piloerection	0–1	0 (0/0)	0 (0/0)	1
14	Palpebral closure	0–2	0 (0/0)	0 (0/0)	1
15	Startle response	0–3	1 (1/1)	1 (1/1)	1
16	Gait	0–3	0 (0/0)	0 (0/0)	1
17	Pelvic elevation	0–3	2 (2/2)	2 (2/2)	1
18	Tail elevation	0–2	1 (1/1)	1 (1/1)	1
19	Touch escape	0–3	2 (2/2)	3 (2/3)	0.003
20	Positional passivity	0–4	0 (0/1)	0 (0/0)	0.8
21	Trunk curl	0–1	1 (1/1)	1 (1/1)	1
22	Limb grasping	0–1	1 (1/1)	1 (1/1)	1
23	Visual placing	0–4	2 (2/3)	2 (2/2)	0.99
24	Grip strength	0–4	2 (1/3)	3 (2/3)	0.018
25	Body tone	0–2	1 (1/1)	1 (1/1)	1
26	Pinna reflex	0–2	1 (1/1)	1 (1/1)	1
27	Corneal reflex	0–2	1 (1/2)	1 (1/1)	0.68
28	Toe pinch	0–4	2 (1/3)	3 (2/3)	0.05
31	Lacrimation	0–1	0 (0/0)	0 (0/0)	1
32	Whisker morphology	0–1	0 (0/0)	0 (0/0)	1
33	Provoked biting	0–1	0 (0/0)	0 (0/1)	0.99
34	Salivation	0–2	0 (0/0)	0 (0/0)	1
35	Heart rate	0–2	1 (1/1)	1 (1/1)	1
36	Abdominal tone	0–2	1 (1/1)	1 (1/1)	1
37	Skin color	0–2	1 (1/1)	1 (1/1)	1
38	Limb tone	0–4	1 (1/2)	1 (1/2)	1
39	Wire maneuver	0–4	0 (0/1)	0 (0/1)	0.8
40	Righting reflex	0–3	0 (0/0)	0 (0/0)	1
41	Contact reflex	0–1	1 (1/1)	1 (1/1)	1
42	Negative geotaxis	0–4	0 (0/0)	0 (0/0)	0.22
43	Fear to experimenter	0–1	0 (0/0)	0 (0/0)	1
44	Irritability	0–1	0 (0/0)	0 (0/0)	1
45	Aggression	0–1	0 (0/0)	0 (0/0)	1
46	Vocalization	0–1	1 (0/1)	1 (0/1)	0.88
47	Bizarre behavior	0–1	0 (0/0)	0 (0/0)	1
48	Weight	0-30	24.8 (22/26)	23 (21/24)	0.14

B. Female

No.	Variable	Scale Range	Control	Anti-Caspr2	*P*
1	Coat length	0–4	0 (0/0)	0 (0/0)	1
2	Hair length	0–3	0 (0/0)	0 (0/0)	1
3	Hair morphology	0–2	0 (0/0)	0 (0/0)	1
4	Body position	0–8	4 (4/5)	5 (4/5)	0.68
5	Spontaneous activity	0–8	5 (4/6)	4 (4/5)	0.05
6	Respiration rate	0–3	2 (2/2)	2 (2/2)	1
7	Tremor	0–2	0 (0/0)	0 (0/0)	1
8	Defecation	0–10	0 (0/2)	0 (0/3)	0.9
9	Urination	0–1	0 (0/0)	0 (0/0)	1
10	Transfer arousal	0–6	4.5 (4/5)	5 (3/5)	1
11	Latency to move	0–30	2.5 (0/3)	0(2/6)	0.26
12	Locomotion	0–30	17(14/23)	18 (11/24)	0.98
13	Piloerection	0–1	0 (0/0)	0 (0/0)	1
14	Palpebral closure	0–2	0 (0/0)	0 (0/0)	1
15	Startle response	0–3	1 (1/1)	1 (1/1)	1
16	Gait	0–3	0 (0/0)	0 (0/0)	1
17	Pelvic elevation	0–3	2 (2/2)	2 (2/2)	1
18	Tail elevation	0–2	1 (1/1)	1 (1/1)	1
19	Touch escape	0–3	3 (2/2)	2 (2/3)	0.01
20	Positional passivity	0–4	0 (0/1)	0 (0/0)	0.45
21	Trunk curl	0–1	1 (1/1)	1 (1/1)	1
22	Limb grasping	0–1	1 (1/1)	1 (1/1)	1
23	Visual placing	0–4	2 (1/3)	2 (2/3)	0.45
24	Grip strength	0–4	2 (1/3)	2 (2/3)	0.005
25	Body tone	0–2	1 (1/1)	1 (1/1)	1
26	Pinna reflex	0–2	1 (1/1)	1 (1/1)	1
27	Corneal reflex	0–2	1 (1/1)	1 (1/1)	1
28	Toe pinch	0–4	2 (2/2)	2 (2/2)	1
31	Lacrimation	0–1	0 (0/0)	0 (0/0)	1
32	Whisker morphology	0–1	0 (0/0)	0 (0/0)	1
33	Provoked biting	0–1	0 (0/0)	0 (0/1)	0.99
34	Salivation	0–2	0 (0/0)	0 (0/0)	1
35	Heart rate	0–2	1 (1/1)	1 (1/1)	1
36	Abdominal tone	0–2	1 (1/1)	1 (1/1)	1
37	Skin color	0–2	1 (1/1)	1 (1/1)	1
38	Limb tone	0–4	2 (2/2)	2 (2/2)	0.86
39	Wire maneuver	0–4	0 (0/0)	0 (0/0)	0.67
40	Righting reflex	0–3	0 (0/0)	0 (0/0)	1
41	Contact reflex	0–1	1 (1/1)	1 (1/1)	1
42	Negative geotaxis	0–4	0 (0/0)	0 (0/0)	1
43	Fear to experimenter	0–1	0 (0/0)	0 (0/0)	1
44	Irritability	0–1	0 (0/0)	0 (0/0)	1
45	Aggression	0–1	0 (0/0)	0 (0/0)	1
46	Vocalization	0–1	0 (0/0)	1 (1/0)	0.005
47	Bizarre behavior	0–1	0 (0/0)	0 (0/0)	1
48	Weight	0-30	18.4 (18/20)	19.4 (18.5/21)	0.085

Primary screen was performed on 5-7 week old mice. The variables were compared with the non-parametric Mann-Whitney test, followed by Bonferroni correction. Significant P value, $P<0.001$. The numbers represent as median (min/max).

Supplementary Figure 1: Dams immunized with Caspr2 and their fetuses harbor anti-Caspr2 IgG

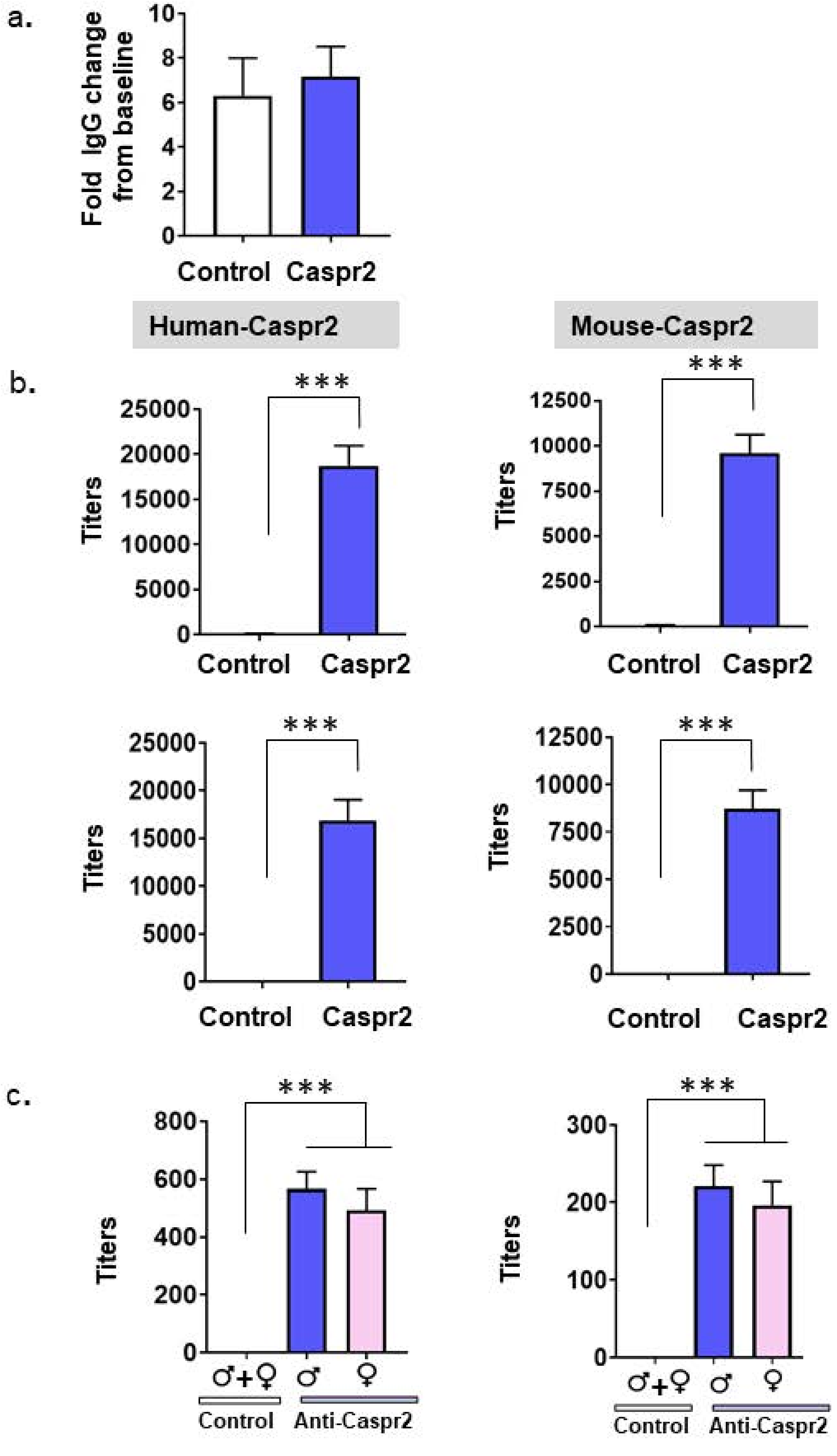

Supplementary Figure 2: No cortical development abnormalities were observed between male fetuses exposed in utero to anti-Caspr2 or Control IgG in Caspr2 -/- mice

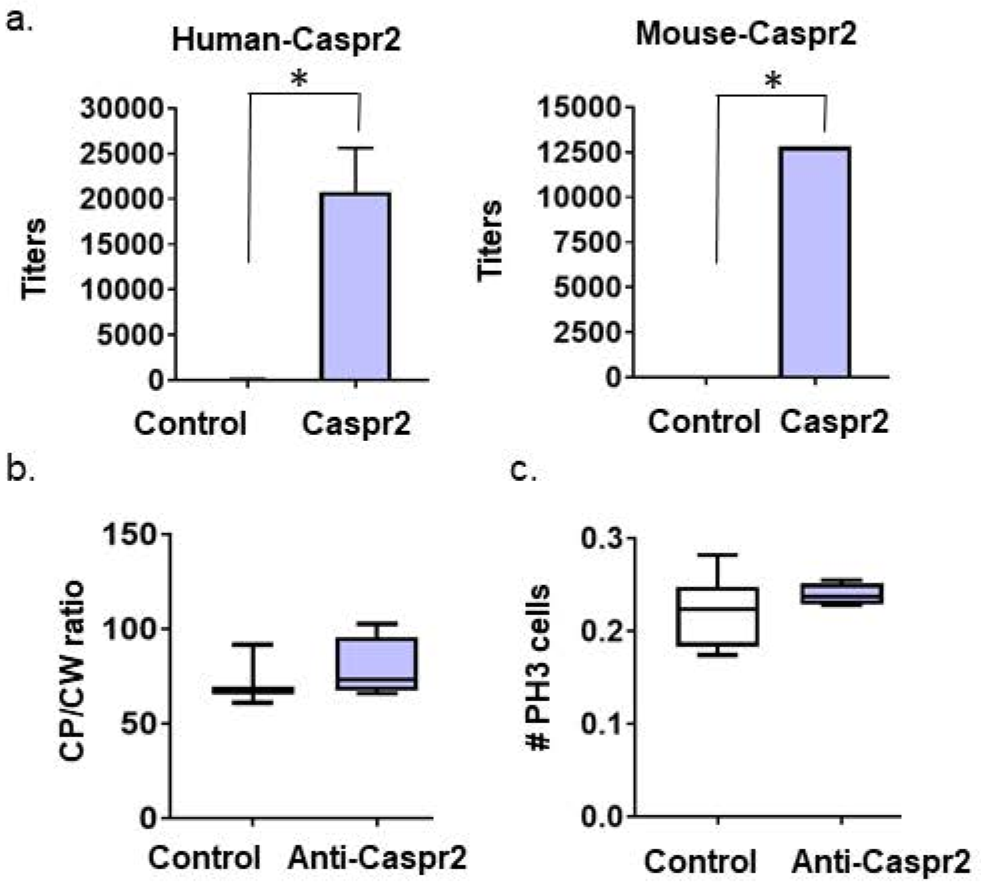

Supplementary Figure 3: Female mice born to dams immunized with Caspr2 showed no abnormality of dendritic arborization in pyramidal neurons and similar number of parvalbumin (PV) GABAergic interneurons compared to Control.

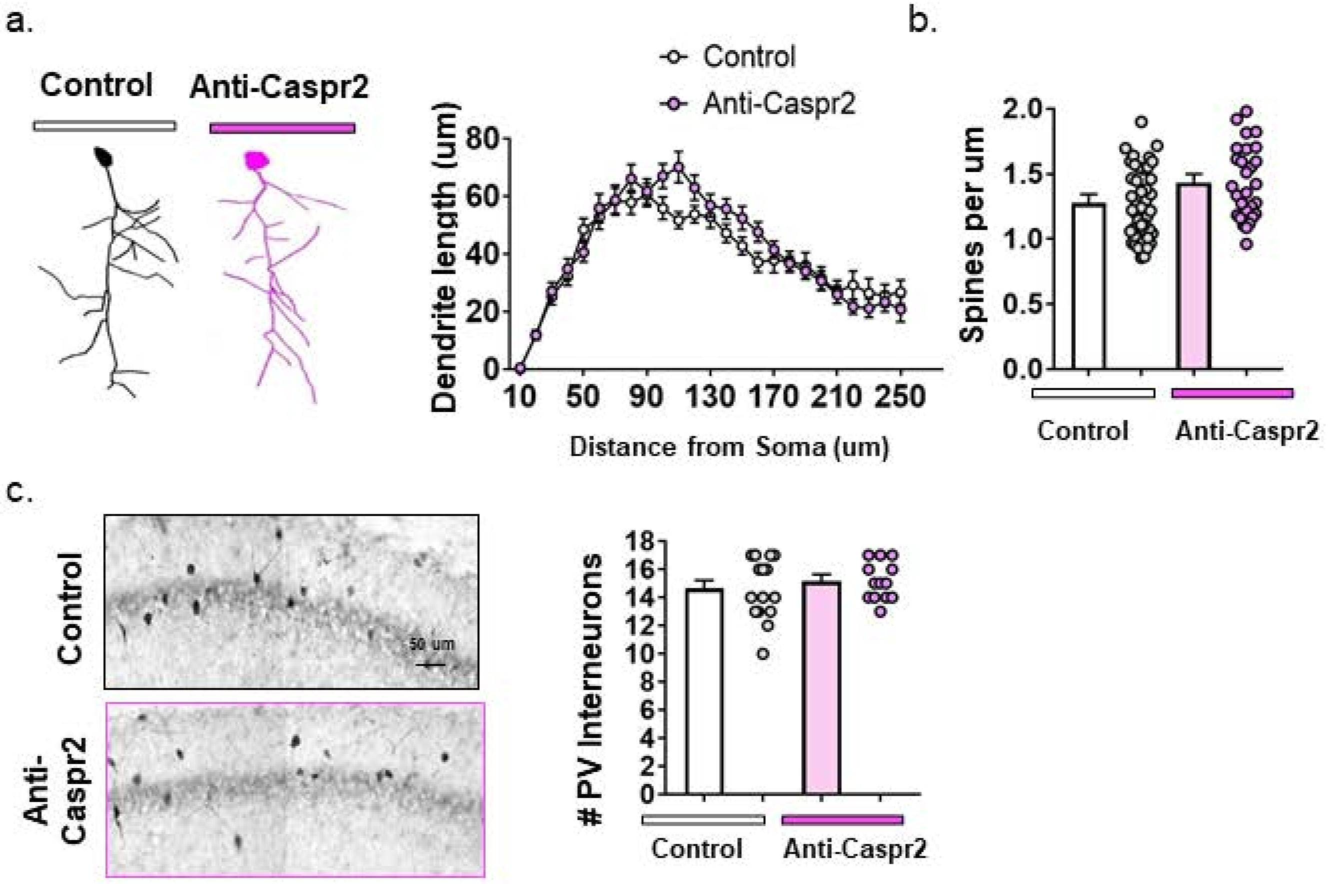

Supplementary Figure 4: Behavioral phenotype of female mice exposed in utero to anti-Caspr2 or Control IgG.

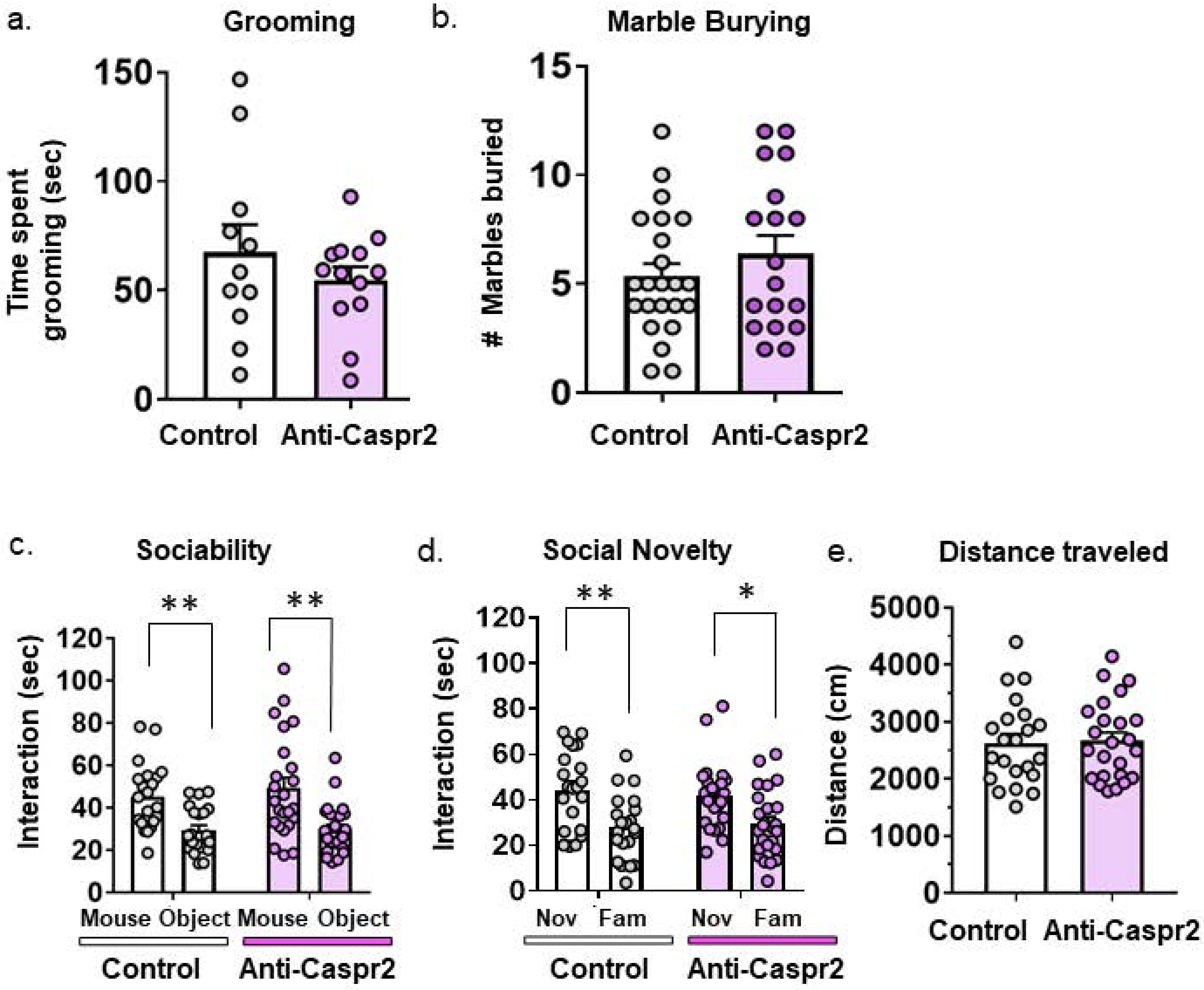

Supplement: Supplementary file 1 [file 41598_2020_71201_MOESM1_ESM.pdf]
